# Metabolic effects of antihyperglycemic agents and mortality: meta-analysis of randomized controlled trials

**DOI:** 10.1038/s41598-020-69738-w

**Published:** 2020-07-30

**Authors:** Dimitris Varvaki Rados, Camila Viecceli, Lana Catani Pinto, Fernando Gerchman, Cristiane Bauermann Leitão, Jorge Luiz Gross

**Affiliations:** 1 Post-Graduate Program in Medical Sciences, Endocrinology, Universidade Federal do Rio Grande do Sul, Rua Ramiro Barcelos 2300, 2º floor, Porto Alegre, RS 90035-903 Brazil; 2 Division of Endocrinology, Hospital de Clínicas de Porto Alegre/Universidade Federal do Rio Grande do Sul, Rua Ramiro Barcelos 2350, Prédio 12, 4º floor, Porto Alegre, RS 90035-903 Brazil

**Keywords:** Type 2 diabetes, Diabetes complications

## Abstract

The effects of antihyperglycemic medications on cardiovascular events and mortality are heterogeneous and their effects on intermediate factors might explain these differences. This systematic review explores the relationship between metabolic factors, mechanism of action, and mortality effects of antihyperglycemic medications in type 2 diabetes. Randomized trials assessing the effects of antihyperglycemic medications on all-cause or cardiovascular mortality in type 2 diabetes were included. Myocardial infarction, stroke, and heart failure were secondary outcomes. The effects of medications on HbA1c, severe hypoglycemia (SH), body weight, systolic blood pressure (SBP), and mechanism of action were evaluated. Meta-analyses and meta-regressions were performed grouping studies according to the above-cited factors. All-cause mortality was lower for medications that reduced HbA1c, SH, body weight, and SBP. Decreased cardiovascular mortality was associated with lower HbA1c, SH, SBP. Myocardial infarction and stroke were also associated with favorable metabolic profile. These findings were not confirmed in meta-regression models. Medications associated with lower SH, body weight and SBP had a lower risk of heart failure. In conclusion, medications with better metabolic profile were associated with reduced all-cause and cardiovascular mortality. These findings are based on indirect comparisons and must be applied cautiously.

## Introduction

Subjects with type 2 diabetes have a high risk of cardiovascular disease, which is the leading cause of death and disability. Many factors influence this risk, such as glucose control, hypoglycemia frequency, body weight, and blood pressure^1–3^.

Unexpectedly, studies analyzing the role of intensified glucose control on cardiovascular mortality were able to achieve significant differences in glycemic control but did not find a reduction in events^4–6^. This lack of benefit may be attributable to the increased number of hypoglycemic events and weight gain associated with strict glycemic control^4–6^.

Several classes of antihyperglycemic medications have been approved for the treatment of type 2 diabetes, but, until recently, metformin was the only medication proven to reduce cardiovascular events and mortality in patients with this condition^7^. In the last years, cardiovascular safety trials showed that some sodium-glucose-linked cotransporter 2 inhibitors (SGLT2i) and glucagon-like peptide-1 receptor agonists (GLP-1RAs) are also capable of reducing cardiovascular events and all-cause mortality in high-risk populations^8–11^. Although these medications have different mechanisms of action and effects on glucose values, they share similar favorable metabolic effects, such as weight loss, and reduced risk of hypoglycemia^12^. Furthermore, the mechanism of action may not be the only determinant of the effects of a medication on cardiac events, as different representatives of the same class seem to have diverse effects on cardiovascular events^8,11,13,14^.

Joint guideline from the European Association for the Study of Diabetes and American Diabetes Association recommend a patient-centered approach^15^. This is based on the selection of medications in type 2 diabetes based on patient factors and considering the heterogeneity of treatment options, such as weight effects, hypoglycemia risk, and previous cardiovascular events^15^. Also, medications are classified as pharmacological classes, and not according to individual representatives or their effects on the above factors^15^. Whether this approach leads to better outcomes to patients is unknown and is mainly based on expert’s opinion.

Considering the heterogeneity between antihyperglycemic agents, we hypothesized that antihyperglycemic medications that lead to better glycemic control, lower severe hypoglycemia risk, lower body weight, and lower blood pressure might reduce the risk of death (all-cause or cardiovascular). We also explored if these outcomes are influenced by the mechanism of action of the medications. The objective of this systematic review with meta-analysis is to evaluate the relationship between the above factors, the treatment with antihyperglycemic medications in type 2 diabetic subjects, and mortality and cardiovascular events.

## Methods

We registered this review and meta-analysis in the International Prospective Register of Systematic Reviews (PROSPERO) under number CRD42016043895. This report follows the PRISMA statement for systematic reviews^16^. Ethical approval was exempted.

### Eligibility, data sources, and searches

Studies were eligible if were performed in subjects with type 2 diabetes (*patients*), evaluated any antihyperglycemic medication (*intervention*), reported all-cause or cardiovascular mortality (*outcome*) and were randomized controlled parallel trials (*study*); no specific comparator was defined. We searched PubMed, EMBASE, the Cochrane Library, and clinicaltrials.org from inception up to May 2020 for randomized controlled trials, performed in patients with type 2 diabetes, that reported any of the main outcomes (all-cause or cardiovascular mortality). There was no additional restriction on the searches. The search terms were *type 2 diabetes AND mortality OR cardiovascular mortality AND randomized controlled trial*. A hand search of reference lists of previous systematic reviews and key articles was also performed, and all potentially eligible studies were considered for review.

### Study selection

Two investigators (DVR and CV) independently screened potentially relevant studies based on titles and abstracts. Studies that met inclusion criteria were thoroughly reviewed. Consensus resolved disagreements. Articles were included if they were randomized controlled trials, included antihyperglycemic medications approved for the treatment of type 2 diabetes, had lasted more than one year and reported at least one of the outcomes of interest (all-cause or cardiovascular mortality).

### Data extraction

Two investigators (DVR and CV) independently extracted relevant data from studies. Change in glycated hemoglobin (HbA1c), body weight, systolic blood pressure, and severe hypoglycemia events, were also extracted. We evaluated all-cause mortality and cardiovascular mortality as primary outcomes and the incidence of cardiovascular events (acute myocardial infarction, stroke, and heart failure) as secondary outcomes. As recommended, when the study comprised more than two groups, we either (a) spliced the intervention group to compare with each of the control groups; or (b) combined the control groups to compare with the intervention group, to perform pair-wise comparisons^17^. Discrepancies in extracted data were resolved by consensus.

### Quality assessment

The Cochrane Collaboration tool was used to assess the bias risk of individual studies^18^. Using the Grading of Recommendations, Assessment, Development and Evaluations (GRADE) method, we ranked the quality of the evidence of each outcome as high, moderate, low, or very low^17^. Summary of findings table was constructed using GRADEPRO software^19^.

### Data synthesis and analysis

To analyze the effects of the factors (glycemic control, severe hypoglycemia risk, body weight, and blood pressure changes) on the outcomes of interest, we adopted the following approach. First, we extracted each variable numerical change from baseline to end of follow-up in the experimental arm. Second, we also extracted the differences between study arms (variable delta of the intervention group minus variable delta of the control group). At last, the effects of an experimental intervention in the factor was classified as “reduction” of the factor (HbA1c, body weight or blood pressure) or “no reduction”. The variable was classified as “reduction” if both changes (variation during follow-up in the experimental arm and between experimental and control arms) were equal or greater than: (a) 0.3% for HbA1c; (b) 1 kg for body weight or; (c) 1 mmHg for systolic blood pressure. We opted this approach as it allows to select interventions that showed real improvements in glycemic control, body weight, or systolic blood pressure, and not a deterioration in the experimental arm that was only smaller than the observed in control group. For example, in UKPDS33 patients in the sulfonylurea group had a mean body weight gain of 2.15 kg, and in the insulin group patients gained 4 kg^20^. With this approach, we avoided classifying this difference as “weigh reduction”. For severe hypoglycemia, the absolute risk difference between intervention and control arms was used. These categories (“reduction” and “no reduction”) were used to stratify the meta-analyses. As an additional and exploratory analysis, we evaluated if the mechanism of action would influence the results and medications were classified as: (a) insulin / secretagogues; (b) insulin-sensitizing; (c) incretins and; (d) SGLT2i.

The outcomes were combined with Mantel–Haenszel relative risks (RR) with random-effects model^17^. We evaluated statistical heterogeneity with Cochran’s Q and the I^2^ test (*P* < 0.1 and I^2^ > 50% indicated elevated heterogeneity, respectively). Small study bias was assessed with the funnel plot asymmetry and with the Begg and Egger tests; if bias was identified, we performed a trim-and-fill computation to evaluate the potential effect of unpublished studies on the results^21,22^. Our objective involved the evaluation and comparison of subgroup analyses in meta-analysis. We followed Cochrane Handbook recommendations for comparing subgroups in random effects model, besides performing stratified analyses, we also compared the subgroups (factors and mechanisms of action) with metaregressions using dichotomous variables^17,23^. Analyses were performed using Stata 13.0 software (StataCorp, College Station, USA). Graphics were constructed with *forestplot* package using Rstudio program.

## Results

### Database review and characteristics of selected studies

**Figure 1:**
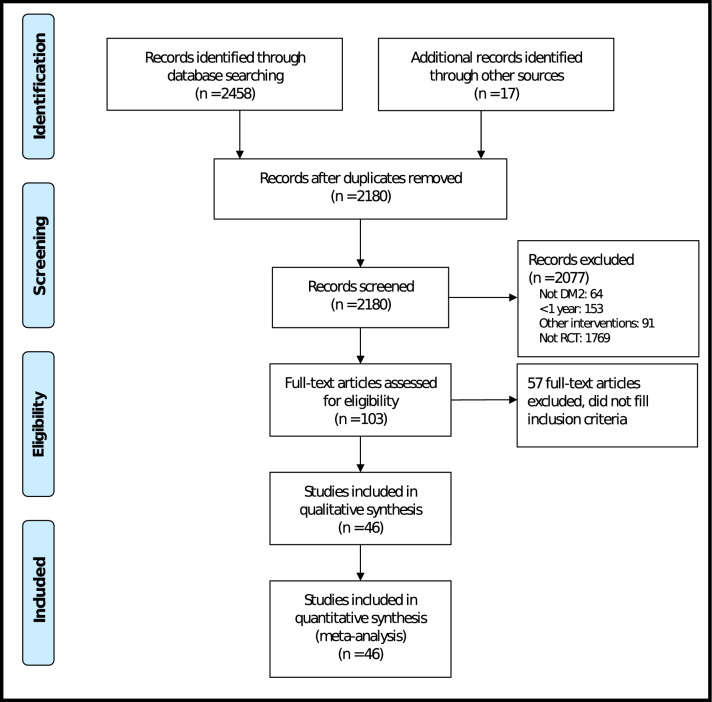
Studies flowchart.

Electronic and manual searches retrieved 2,180 potential studies, of which 2077 were excluded based on titles and abstracts and 103 were selected for full-text review. In total, 46 studies met the inclusion criteria. A complete flow diagram of study inclusion is shown in Fig. 1.

The included studies represent a total of 216,575 patients and a total of 15,304 all-cause deaths and 8,994 cardiovascular deaths. Summarized study characteristics are presented in Table 1; detailed characteristics of the included studies are presented in Table S1 and S2 (Supplementary material). Studies evaluated the effects of almost every class of antihyperglycemic agents; incretin-based therapies were the most common medications assessed (thirteen studies of dipeptidyl peptidase-4 inhibitors and eight studies of GLP-1RA). About half of the studies (24 from 46) had active comparators, of which sulfonylurea was the predominant class.

**Table 1:** Studies characteristics. N.A. = not available; CV = cardiovascular.

First author	Year	Study drug -experimental	Study drug -control	Age (years)	Previous CV event	Number of patients	HbA1c difference (%)	Severe hypoglycemia difference (%)	Weight difference (kg)	Systolic blood pressure difference (mmHg)	Follow-up (years)
Turner^7^	1998	Intensive with metformin	Intensive with insulin or sulfonylurea	53	N.A	1,293	No reduction	Reduction	No reduction	N.A	10.7
Turner^20^	1998	Intensive with sulfonylurea	Intensive with insulin	54	N.A	2,145	No reduction	N.A	No reduction	N.A	10
Dormandy^41^	2005	Pioglitazone		61.9	100%	5,238	Reduction	No reduction	No reduction	N.A	2.8
Kahn^24^	2006	Metformin	Rosiglitazone	57.9	N.A	2,183	No reduction	No reduction	Reduction	N.A	4
Kahn^24^	2006	Metformin	Glibenclamide	57.9	N.A	2,168	No reduction	Reduction	Reduction	N.A	4
Mazzone^42^	2006	Pioglitazone	Glimepiride	59.3	N.A	458	Reduction	Reduction	No reduction	No reduction	1.5
Nauck^43^	2006	Sitagliptin	Glipizide	56.8	N.A	1,172	No reduction	Reduction	Reduction	N.A	1
Dargie^44^	2007	Rosiglitazone		64.3	N.A	224	Reduction	N.A	No reduction	N.A	1
Chan^45^	2008	Sitagliptin	Glipizide	68.9	44%	91	No reduction	Reduction	No reduction	N.A	4.5
Patel^6^	2008	intesive glucose control	Conventional glucose control	66	32%	11,140	N.A	No reduction	N.A	N.A	5
Holman^26^	2009	Basal-bolus or bolus only	Basal only	61.7	N.A	708	No reduction	No reduction	No reduction	No reduction	3
Home^46^	2009	Rosiglitazone	Metformin or Sulfonylureia	58.4	32%	4,447	No reduction	No reduction	No reduction	No reduction	5.5
Kooy^47^	2009	Metformin		64	N.A	390	No reduction	N.A	No reduction	Reduction	4.3
Bertrand^48^	2010	Rosiglitazone		64.2	N.A	193	Reduction	N.A	No reduction	No reduction	1
Gaziano^49^	2010	Bromocriptin QR		59.5	37%	3,070	No reduction	N.A	No reduction	Reduction	1
Giles^50^	2010	Pioglitazone	Glibenclamide	64	N.A	300	No reduction	N.A	No reduction	No reduction	1
Matthews^51^	2010	Vildagliptin	Glimepiride	57.5	N.A	3,118	No reduction	N.A	No reduction	N.A	2
Gallwitz^52^	2012	Exenatide	Glimepiride	56	N.A	977	No reduction	No reduction	Reduction	Reduction	3
Gallwitz^53^	2012	Linagliptin	Glimepiride	59.8	N.A	1551	No reduction	Reduction	Reduction	N.A	2
Garber^54^	2012	Degludec	Glargine	59.2	N.A	992	No reduction	No reduction	No reduction	N.A	1
Gerstein^55^	2012	Glargine	Control	64.5	59%	12,537	No reduction	No reduction	No reduction	No reduction	6.2
Zinman^56^	2012	Degludec	Glargine	59.3	N.A	1,030	No reduction	Reduction	No reduction	N.A	1
Cefalu^57^	2013	Canagliflozin	Glimepiride	56.1	N.A	1,450	No reduction	Reduction	Reduction	Reduction	1
Hong^58^	2013	Metformin	Glipizide	62.8	100%	304	No reduction	N.A	Reduction	Reduction	3
Scirica^59^	2013	Saxagliptin		65.1	79%	16,492	No reduction	No reduction	No reduction	N.A	2.1
White^60^	2013	Alogliptin		61	100%	5,380	Reduction	No reduction	No reduction	N.A	1.5
Ridderstrale^61^	2014	Empagliflozin	Glimepiride	56.2	N.A	1545	No reduction	N.A	Reduction	Reduction	2
Blonde^62^	2015	Dulaglutide	Glargine	59.1	N.A	884	No reduction	Reduction	No reduction	No reduction	1
Giorgino^63^	2015	Dulaglutide	Glargine	56.5	N.A	807	No reduction	Reduction	Reduction	No reduction	1.5
Green^64^	2015	Sitagliptin		65.4	74%	14,671	N.A	No reduction	N.A	N.A	3
Pfeffer^13^	2015	Lixisenatide		59.9	100%	6,068	Reduction	Reduction	No reduction	No reduction	2.1
Zinman^9^	2015	Empagliflozin		63.1	100%	7,020	Reduction	Reduction	No reduction	No reduction	3.1
Marso^11^	2016	Semaglutide		64.6	N.A	9,340	Reduction	Reduction	Reduction	Reduction	3.8
Marso^8^	2016	Liraglutide		64.2	100%	3,297	Reduction	N.A	Reduction	Reduction	2
Holman^65^	2017	Exenatide		62	73%	14,752	Reduction	No reduction	No reduction	Reduction	3.2
Marso^66^	2017	Degludec	Glargine	64.9	63%	7,637	No reduction	Reduction	No reduction	No reduction	1.99
Neal^10^	2017	Canagliflozin		63.2	66%	10,142	Reduction	N.A	Reduction	Reduction	3.6
Vaccaro^67^	2017	Pioglitazone	Glimepiride	62.4	11%	3,028	No reduction	Reduction	No reduction	No reduction	4.7
Hernndez^68^	2018	Albiglutide		64.15	100%	9,463	Reduction	Reduction	No reduction	No reduction	1.6
Rosenstock^69^	2018	Linagliptin		65.85	59%	6,979	N.A	Reduction	No reduction	No reduction	2.2
Gerstein^70^	2019	Dulaglutide		66.2	31%	9,901	Reduction	Reduction	Reduction	Reduction	5.4
Husain^71^	2019	Oral Semaglutide		66	85%	3,183	Reduction	No reduction	Reduction	Reduction	1.3
Perkovic^72^	2019	Canagliflozin		63.05	50%	4,401	No reduction	N.A	No reduction	Reduction	2.62
Pieber^73^	2019	Oral Semaglutide	Sitagliptin	57.9	N.A	504	Reduction	No reduction	Reduction	N.A	1
Pratley^25^	2019	Oral Semaglutide		56	N.A	356	Reduction	Reduction	Reduction	Reduction	1
Pratley^25^	2019	Liraglutide		56	N.A	355	Reduction	Reduction	Reduction	Reduction	1
Wiviott^74^	2019	Dapagliflozin		63.95	41%	17,160	Reduction	Reduction	Reduction	Reduction	4.2
Rosenstock^75^	2019	Linagliptin	Glimepiride	64.1	34%	6,033	No reduction	Reduction	Reduction	No reduction	6.3

Three studies had more than two arms and needed group splicing or combining to be included in this meta-analysis. In the ADOPT and PIONEER 4 studies^24,25^, we spliced one study arm (ADOPT: metformin; PIONEER 4: placebo) and compared it to remainder arms. In the 4-T study^26^, we combined the groups receiving bolus insulin (prandial insulin and biphasic insulin).

Overall, the risk of bias was low, especially for randomization and blinding. For incomplete outcome data, six studies had high risk of bias (mostly due to an unbalanced number of patients without follow-up information). Detailed assessment information on the risk of bias of individual studies is presented in the supplementary appendix (Table S3, Supplementary material).

### Main results

**Figure 2:**
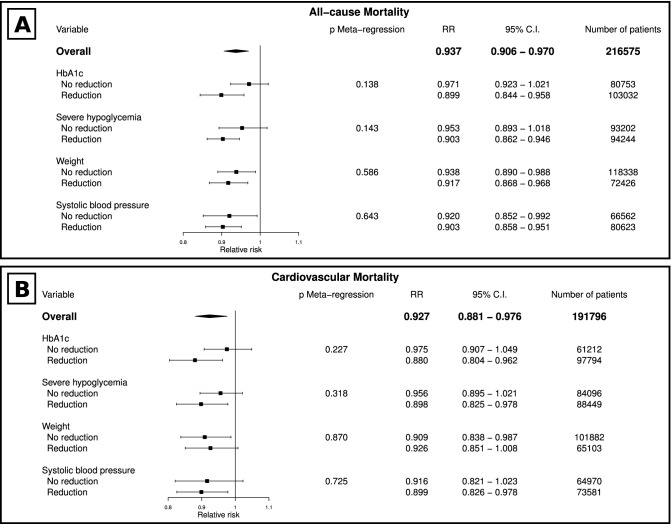
Forest plot for antihyperglycemic agents and all-cause and cardiovascular mortality relative risks according to glycemic control, severe hypoglycemia risk, weight variation, and systolic blood pressure variation. Legend: (**A**) all-cause mortality; (**B**) cardiovascular mortality.

As shown in Fig. 2 and Table S4 (Supplementary material), antihyperglycemic medications are associated with reduced risk of all-cause and cardiovascular mortality overall. Stratifying the analysis according to glycemic control, severe hypoglycemia risk, body weight, and systolic blood pressure showed that treatments that lead to reduction of these factors (except weight change for cardiovascular mortality) were associated with lower all-cause mortality. Glycemic control was the factor with the greatest numeric reduction of all-cause mortality (RR 0.90 [95% CI 0.84–0.96]) and cardiovascular mortality (RR 0.88 [95% CI 0.80–0.96]); Table S3, Supplementary material. In the meta-regression models, none of these comparisons were statistically significant.

Regarding analyses evaluating mechanisms of action of medications (Table S4 and Figure S1A and S1B, Supplementary material), only incretins were associated with reduced all-cause or cardiovascular mortality. Meta-regression models found that this difference was not statistically significant.

Statistical heterogeneity was low (Table S4, Supplementary material). Small study bias was not identified with Egger and Begg tests and funnel plot inspection (Supplementary material, Figure S2 A and B).

### Secondary outcomes

Treatments that lead to lower HbA1c, severe hypoglycemia risk, body weight, and systolic blood pressure were associated with a lower risk for myocardial infarction (Table S4 and Figures S1C and S1D, Supplementary material). Meta-regressions did not confirm these findings. Statistical heterogeneity was low. Small study bias risk was detected for myocardial infarction (Figure S2C, Supplementary material), but trim and fill computation did not change the results.

Reduction of HbA1c, body weight, and systolic blood pressure was associated with lower stroke risk (Table S4 and Figures S1E and S1F, Supplementary material). Meta-regression analyses also did not confirm these findings. Statistical heterogeneity was low, and small study bias was not identified (Figure S2D, Supplementary material). Insulin sensitizers and incretins were associated with lower stroke risk. Although meta-regression did not confirm these associations, for insulin sensitizers the results were borderline (*P* = 0.055).

As shown in Table S4 and Figures S1G and S1H (Supplementary material), reduction of severe hypoglycemia risk, body weight, and systolic blood pressure factors were associated with a lower risk of heart failure. None of this factors were confirmed in meta-regression. Of note, statistical heterogeneity was elevated in heart failure analyses. Small study bias was not identified (Figure S2E, Supplementary material). Regarding mechanism of action, insulin sensitizers were associated with a higher risk of heart failure and SGLT2i were associated with lower risk, both in stratified and meta-regression analyses.

### Grading quality of evidence

The GRADE quality of evidence for all outcomes was moderate and the summary of findings are presented in supplementary material (Table S5, Supplementary material). Despite being based on high-quality studies with low risk of bias, our observations on the effects of clinical factors on the outcomes of interest (all-cause and cardiovascular mortality, myocardial infarction, stroke, and heart failure) are indirect due to the nature of the analysis^17^.

## Discussion

Based on data from high-quality RCTs, we found that antihyperglycemic agents that lead to lower HbA1c, severe hypoglycemia risk, body weight, and systolic blood pressure were associated with reduced risk of all-cause and myocardial infarction in patients with type 2 diabetes. Cardiovascular mortality was associated with the same factors except body weight. As the analyses are indirect, based on aggregated patient data and meta-regression models did not confirm the findings, this information must be applied and generalized cautiously.

The results for secondary outcomes are also interesting. The association of SGLT2i with reduced risk for heart failure and insulin sensitizers with a higher risk for heart failure has been already shown^27^. However, the relation between severe hypoglycemia and heart failure is novel. The mechanism may be a true effect of hypoglycemia on myocardial cells^28^, however it is more likely to represent medications with safer cardiovascular profile. The association between insulin sensitizers and stroke has been observed previously^7,29^. Lower insulin resistance may be the promoter of this benefit, and numerically has a greater effect on stroke than myocardial infarction risk^30^.

These findings are based on an extensive and systematic literature review. The studies identified had mostly low risk of bias. This allowed us to explore our objective with high quality data and with a large number of patients. The definition of “reduction” of a metabolic factor combined the differences within and between arms to improve confidence in the classification. We also followed the recommended statistical approach to compare the subgroups of medications. These complementary analyses allowed the identification of the limitations of our data.

Previous systematic reviews with meta-analysis explored the effects of different antihyperglycemic medications on mortality or cardiovascular events. Two recent network meta-analysis showed contradictory results: one failed to identify evident superiority of any drug class, and the other found that SGLT2i and GLP-1RAs were associated with reduced risk of cardiovascular events and mortality^31,32^. We believe these results reinforce the importance of the hypothesis explored in our systematic review. Individually, several medication classes were assessed for benefit or harm in systematic reviews of RCTs and also failed to show a consistent effect on mortality and cardiovascular events^33–36^. Our review broadens these findings and explored the relationship between metabolic profile and mechanism of action of these medications and mortality. In the stratified meta-analyses, our findings suggest that the metabolic effects of medications must be considered in treatment selection of patients with type 2 diabetes. Also, these results agree with current guidelines, that recommend considering other factors besides the simple glycemic effects of antihyperglycemic agents^15^.

This is the first systematic review exploring this topic, but some primary studies evaluated this research question recently. An observational study from a Swedish database showed that glycated hemoglobin, systolic blood pressure, and body mass index are all important risk factors for all-cause mortality in patients with type 2 diabetes^37^. Individual data from large RCTs also indicate the same pattern. An additional analysis of Look AHEAD indicates that patients in the category of greatest weight loss in the first year had decreased risk of cardiovascular death and non-fatal cardiovascular events independent from the arm they were originally randomized^38^. This Look AHEAD analysis, which corroborates our findings, reinforces that diabetes treatments must focus on other targets (such as avoidance of hypoglycemia, lower weight gain, and lower blood pressure) in addition to glycemic control. Data from EMPA-REG OUTCOME trial suggest that plasma volume contraction may be the main determinant of benefits from empagliflozin treatment^39^. However, glycemic control, systolic blood pressure, and body weight were also associated with a lower risk of cardiovascular death^39^. These studies reinforce the reliability of our findings. Further analyses of primary data from RCTs (individually or through individual patient data meta-analysis) could also provide valuable information^17,40^.

From a mechanistic point of view, our results are biologically plausible, as better glycemic control, less severe hypoglycemic events, lower weight and blood pressure were all associated with better outcomes in subjects with type 2 diabetes in observational or interventional studies^1–3^. However, this systematic review does not determine if: (a) the factors are predictors of a beneficial effect of these medications or; (b) there is a mechanistic relationship between favorable overall medication profile and mortality and cardiovascular events. As we observed small changes in the metabolic factors, it seems more likely that there is association rather than causation. That is, small changes on intermediate outcomes (glycemic control, severe hypoglycemia risk, body weight, and blood pressure) are not expected to be the determinants of reduced mortality risk. We also could not explore the effects of medications on cholesterol, as reporting was available only in a few studies.

This study has some limitations inherent to the design used. We aimed to explore the new hypothesis that other factors along with mechanism of action of each antihyperglycemic agent might explain the variability on its clinical effects. Cochrane Handbook states that subgroup analyses are observational^17^, so our findings must be considered indirect. They are susceptible to bias, such as confounding. Our results represent an association between the study variables and outcomes; so they may not represent causation. Associations between metabolic factors, mechanism of action, and outcomes must be confirmed, as most of the meta-regressions did not explain the heterogeneity within study results. However, we cannot assume the lack of association between studied factors and outcomes only based on meta-regression results as “one should never use a nonsignificant finding to conclude that the true means in subgroups are the same, or that a covariate is not related to effect size”^23^. Also, as this meta-analysis deals with study-level characteristics, only summarized effects of each study are evaluated and relevant association between factors and outcomes may be missed^17^. In other words, the link between “better metabolic profile” of antihyperglycemic medications and reduced mortality must be further explored and studied, ideally with meta-analysis from individual patient data^17,40^.

Another issue of this review is the combination of different classes of medications. This problem may be considered the main limitation as there is unquestionable clinical heterogeneity in this approach. However, we explored this limitation by considering the mechanisms of action of medication in our analyses. Also, this limitation is inherent to addressing the hypothesis that determinants other than the mechanism of action of antihyperglycemic agents may mediate the clinical effects of antihyperglycemic agents. The use of different comparators is a similar problem. We tried to partially control this limitation by using the differences between study arms rather than using the absolute values. Network meta-analysis might deal with the problem of different comparators. However, it does not allow the evaluation of co-factors and we would not be able to explore our hypothesis.

## Conclusion

Antihyperglycemic medications that lead to better overall metabolic profile were associated with decreased all-cause and cardiovascular mortality in patients with type 2 diabetes. Our results were based on indirect comparisons of study-level information and were not confirmed in meta-regression analyses, so this topic must be further studied. Although this data must be considered preliminary, it is relevant to patient and clinicians in the choice of antihyperglycemic treatment in type 2 diabetes.

## Supplementary information

Supplementary file1 (PDF 673 kb)

## Data Availability

All relevant data are within the paper and the supplementary material.
