# Supplementary material for: Metabolic effects of antihyperglycemic agents and mortality: meta-analysis of randomized controlled trials
Source: Sci Rep. 2020 Jul 30;10:12837. doi: 10.1038/s41598-020-69738-w (PMC7393357; doi:10.1038/s41598-020-69738-w)
Supplement: Supplementary file 1 — Supplementary file1 (PDF 673 kb) [file 41598_2020_69738_MOESM1_ESM.pdf]

Metabolic effects of antihyperglycemic agents and mortality: meta-analysis of randomized controlled trials

Authors: Dimitris Varvaki Rados, Camila Viecceli, Lana Catani Pinto, Fernando Gerchman, Cristiane Bauermann Leitão, Jorge Luiz Gross

Supplementary material

Table S1. Detailed metabolic factors.

First Author	Year	Study Name	Study registry number	HbA1c difference within experimental arm (%)*	Weight difference within experimental arm (kg)*	SBP difference within experimental arm (kg)*	HbA1c difference between study arms (%)#	Severe hypoglycemia difference between study arms (%)#	Weight difference between study arms (kg)#	SBP difference between study arms (mmHg)#
Turner, R	1998	UKPDS 34	N.A.	0.4	2.41	N.A.	-0.9	-0.89%	-3.26	N.A.
Turner, R	1998	UKPDS 33	N.A.	0.65	2.15	N.A.	-0.35	N.A.	-1.85	N.A.
Dormandy, HA	2005	PROACTIVE	174993	-0.8	3.6	N.A.	-0.5	0.31%	3.2	N.A.
Kahn, SE	2006	ADOPT - R	279045	-0.2	-2.9	N.A.	-0.2	0.07%	-7.74	N.A.
Kahn, SE	2006	ADOPT - G	279045	-0.2	-2.9	N.A.	-0.525	-0.42%	-4.47	N.A.
Mazzone, T	2006	CHICAGO	225264	-0.4	3.2	2	-0.32	-7.69%	2.2	0.3
Nauck, MA	2006	N.A.	94770	-0.51	-1.5	N.A.	0.04	-1.20%	-2.5	N.A.
Dargie, HJ	2007	N.A.	N.A.	-0.5	1.3	N.A.	-0.65	N.A.	1.6	N.A.
Chan, JC	2008	N.A.	N.A.	-0.7	-0.9	N.A.	0.3	N.A.	-0.9	N.A.
Patel, A	2008	ADVANCE	145925		N.A.	N.A.	-0.75	1.24%	0.9	-2.4
Holman, RR	2009	4-T	51125379	-1.35	6.04	0.15	-0.15	1.47%	2.44	-0.35
Home, PD	2009	RECORD	379769	-0.36	3.9	-1.5	-0.28	0.41%	3.15	0.04
Kooy, A	2009	HOME	375388	-0.2	2	-19	-0.2	N.A.	-2	-1
Bertrand, OF	2010	VICTORY	169832	-0.5	2.9	4	-0.6	N.A.	2.5	1
Gaziano, JM	2010	N.A.	377676	0.1	0.2	-2	-0.1	N.A.	0.1	-2
Giles, TD	2010	N.A.	521742	-0.8	2.56	1.4	-0.04	N.A.	1.7	1.6
Matthews, DR	2010	N.A.	N.A.	-0.1	-0.3	N.A.	0	N.A.	-1.5	N.A.
Gallwitz, B	2012	EUREXA	359762	-0.37	-3.32	-1.9	-0.17	0.20%	-4.47	-3
Gallwitz, B	2012	N.A.	622284	-0.21	-1.4	N.A.	0.2	-1.42%	-2.7	N.A.

Garber, AJ	2012	BEGIN BB T2D	972283	-1.18	3.6	N.A.	0.07	0.13%	-0.4	N.A.
Gerstein, HC	2012	ORIGIN	69784	-0.2	1.6	-1	-0.3	3.65%	2.1	4
Zinman, B	2012	BEGIN Once Long	765817	-1.06	2.4	N.A.	0.13	-1.69%	0.3	N.A.
Cefalu, WT	2013	CANTATA-SU	968812	-0.87	-3.8	-3.9	-0.06	-2.60%	-4.5	-3.7
Hong, J	2013	SPREAD-DIMCAD	513630	-0.6	-1	-2.1	-0.1	N.A.	-1.9	-1.6
Scirica, BM	2013	SAVOR-TIMI 53	1107886	-0.3	-0.4	N.A.	-0.2	0.43%	-0.1	N.A.
White, WB	2013	EXAMINE	968708	-0.33	1.09	N.A.	-0.36	0.07%	0.05	N.A.
Ridderstrale, M	2014	N.A.	1167881	-0.66	-3.15	-3.1	-0.11	N.A.	-4.61	-5.6
Blonde, L	2015	AWARD-4	1191268	-1.46	0.95	0.3	-0.23	-2.18%	-2.65	-1.6
Giorgino, F	2015	AWARD-2	1394952	-0.75	-1.59	-0.65	-0.15	-0.40%	-3.04	-1.16
Green, JB	2015	TECOS	790205		N.A.	N.A.	N.A.	0.23%	N.A.	N.A.
Pfeffer. MA	2015	ELIXA	1147250	-0.4	-0.6	2.12	-0.3	-0.33%	-1.1	-0.8
Zinman, B	2015	EMPA-REG OUTCOME	1131676	-1.07	-2.3	-0.74	-0.3	-0.20%	-0.83	-1.12
Marso, SP	2016	SUSTAIN-6	1720446	-1	-3.1	-1.3	-0.8	N.A.	-3.6	-1.9
Marso, SP	2016	LEADER	1179048	-1.2	-4.2	-4.4	-0.3	-0.83%	-2.2	-1.3
Holman, RR	2017	EXSCEL	1144338	-0.63	0	-3.5	-0.53	0.40%	-1.27	-1.57
Marso, SP	2017	DEVOTE	1959529	-0.9	2.2	0	0	-5.03%	0.3	N.A.
Neal, B	2017	CANVAS	1989754	-1.02	-3.95	-5.65	-0.58	N.A.	-1.6	-3.93
Vaccaro, O	2017	TOSCA.IT	700856	-0.43	1.1	0	-0.04	-2.08%	1.4	0
Hernndez, AF	2018	Harmony Outcomes	2465515	-0.9	0.8	1.3	-0.3	-0.51%	0.4	1
Rosenstock, J	2018	CARMELINA	1897532		-0.5	-1	-0.36	-0.07%	0.5	0

Gerstein, HC	2019	REWIND	1394952	-0.46	-2.95	-3.15	-0.61	-0.20%	-1.46	-1.7
Husain, M	2019	PIONEER 6	2692716	-1	-4.2	-5	-0.7	0.63%	-3.4	-2.6
Perkovic, V	2019	CREDENCE	2065791	-0.43	-1.89	-3.73	-0.11	N.A.	-0.8	-2.38
Pieber, TR	2019	PIONEER 7	2849080	-1.3	-2.6	N.A.	-0.5	0.00%	-1.9	N.A.
Pratley, R	2019	PIONEER 4 - S	2863419	-1.2	-4.3	-3	-1	-0.71%	-3.3	-3
Pratley, R	2019	PIONEER 4 - L	2863419	-0.9	-3	-2	-0.7	-0.35%	-2	-2
Wiviott, SD	2019	DECLARE-TIMI 58	1730534	-0.4	-4	-3	-0.42	-0.29%	-1.8	-2.7
Rosenstock, J	2019	CAROLINA	1243424	-0.07	-2.4	-1.1	0.1	-1.8%	-1.4	0

N.A. not available
* Final minus baseline for experimental group
# Experimental minus control group

Table S2. Detailed studies events.

First Author	Year	Study Name	Study registry number	Number of patients - experimental	Number of patients - control	All-cause deaths - arm 1	All-cause deaths - arm 2	CV deaths - arm 1	CV deaths - arm 2	AMI - arm 1	AMI - arm 2	Stroke - arm 1	Stroke - arm 2	Heart failure - arm 1	Heart failure - arm 2
Turner, R	1998	UKPDS 34	N.A.	342	951	50	150	N.A.	N.A.	39	139	12	60	N.A.	N.A.
Turner, R	1998	UKPDS 33	N.A.	1234	911	257	184	N.A.	N.A.	190	149	78	42	46	25
Dormandy, HA	2005	PROACTIVE	174993	2605	2633	177	186	N.A.	N.A.	131	157	86	107	417	302
Kahn, SE	2006	ADOPT - R	279045	727	1456	16	34	N.A.	N.A.	11	25	10	16	10	22
Kahn, SE	2006	ADOPT - G	279045	727	1441	15	31	N.A.	N.A.	10	15	9	17	9	9
Mazzone, T	2006	CHICAGO	225264	230	228	1	0	0	0	0	1	0	1	1	0
Nauck, MA	2006	N.A.	94770	588	584	1	2	0	2	N.A.	N.A.	N.A.	N.A.	N.A.	N.A.
Dargie, HJ	2007	N.A.	N.A.	110	114	8	5	5	4	N.A.	N.A.	N.A.	N.A.	N.A.	N.A.
Chan, JC	2008	N.A.	N.A.	65	26	5	1	4	0	3	0	N.A.	N.A.	5	1
Patel, A	2008	ADVANCE	145925	5571	5569	498	533	253	289	153	156	214	209	220	231
Holman, RR	2009	4-T	51125379	474	234	16	3	N.A.	N.A.	6	6	3	2	10	4
Home, PD	2009	RECORD	379769	2220	2227	136	157	60	71	64	56	46	63	61	29
Kooy, A	2009	HOME	375388	196	194	5	5	3	1	6	7	9	9	3	4
Bertrand, OF	2010	VICTORY	169832	98	95	0	0	0	0	0	1	1	1	N.A.	N.A.
Gaziano, JM	2010	N.A.	377676	2054	1016	4	2	N.A.	N.A.	7	9	5	6	9	6
Giles, TD	2010	N.A.	521742	151	149	2	2	0	2	N.A.	N.A.	N.A.	N.A.	10	6
Matthews, DR	2010	N.A.	N.A.	1562	1556	7	6	N.A.	N.A.	N.A.	N.A.	N.A.	N.A.	N.A.	N.A.
Gallwitz, B	2012	EUREXA	359762	490	487	5	5	N.A.	N.A.	1	2	1	0	1	0

Gallwitz, B	2012	N.A.	622284	776	775	4	4	2	2	6	10	3	11	3	2
Garber, AJ	2012	BEGIN BB T2D	972283	744	248	8	2	5	1	N.A.	N.A.	N.A.	N.A.	N.A.	N.A.
Gerstein, HC	2012	ORIGIN	69784	6264	6273	951	965	580	576	336	326	331	319	310	343
Zinman, B	2012	BEGIN Once Long	765817	773	257	1	1	0	0	N.A.	N.A.	N.A.	N.A.	N.A.	N.A.
Cefalu, WT	2013	CANTATA-SU	968812	968	482	2	2	N.A.	N.A.	N.A.	N.A.	N.A.	N.A.	N.A.	N.A.
Hong, J	2013	SPREAD-DIMCAD	513630	156	148	7	14	7	11	5	6	10	15	9	10
Scirica, BM	2013	SAVOR-TIMI 53	1107886	8280	8212	420	378	269	260	265	278	157	141	289	228
White, WB	2013	EXAMINE	968708	2701	2679	153	173	112	130	187	173	29	32	N.A.	N.A.
Ridderstrale, M	2014	N.A.	1167881	765	780	5	5	N.A.	N.A.	N.A.	N.A.	N.A.	N.A.	N.A.	N.A.
Blonde, L	2015	AWARD-4	1191268	588	296	2	3	N.A.	N.A.	N.A.					
Giorgino, F	2015	AWARD-2	1394952	545	262	1	2	1	1	N.A.	N.A.	N.A.	N.A.	N.A.	N.A.
Green, JB	2015	TECOS	790205	7332	7339	547	537	380	366	300	316	178	183	228	229
Pfeffer, MA	2015	ELIXA	1147250	3034	3034	211	223	88	93	255	247	54	49	122	127
Zinman, B	2015	EMPA-REG OUTCOME	1131676	4687	2333	269	194	172	137	261	141	164	69	126	95
Marso, SP	2016	SUSTAIN-6	1720446	4668	4672	381	447	219	278	292	339	179	199	218	248
Marso, SP	2016	LEADER	1179048	1648	1649	62	60	44	46	47	64	27	44	59	54
Holman, RR	2017	EXSCEL	1144338	7356	7396	507	584	340	383	483	493	189	218	219	231
Marso, SP	2017	DEVOTE	1959529	3818	3819	202	221	136	142	144	169	71	79	N.A.	N.A.
Neal, B	2017	CANVAS	1989754	5795	4347	400	281	268	185	215	159	158	116	123	120
Vaccaro, O	2017	TOSCA.IT	700856	1535	1493	55	50	9	4	21	24	16	20	N.A.	N.A.
Hernndez, AF	2018	Harmony Outcomes	2465515	4731	4732	196	205	122	130	181	240	94	108	N.A.	N.A.

Rosenstock, J	2018	CARMELINA	1897532	3494	3485	367	373	255	264	165	146	81	88	209	226
Gerstein, HC	2019	REWIND	1394952	4949	4952	536	592	317	346	223	231	158	205	213	226
Husain, M	2019	PIONEER 6	2692716	1591	1592	23	45	15	30	37	31	12	16	21	24
Perkovic, V	2019	CREDENCE	2065791	2202	2199	168	201	110	140	N.A.	N.A.	N.A.	N.A.	89	141
Pieber, TR	2019	PIONEER 7	2849080	0	251	0	2	0	2	N.A.	N.A.	N.A.	N.A.	N.A.	N.A.
Pratley, R	2019	PIONEER 4 - S	2863419	285	71	3	1	1	0	1	1	2	1	0	0
Pratley, R	2019	PIONEER 4 - L	2863419	284	71	4	1	1	0	1	1	0	0	0	0
Wiviott, SD	2019	DECLARE-TIMI 58	1730534	8582	8578	529	570	245	249	393	441	235	231	212	286
Rosenstock, J	2019	CAROLINA	1243424	3023	3010	308	336	169	168	153	148	104	120	166	155

N.A. not available

Table S3. Risk of bias of individual studies.

	Sequence generation	Allocation concealment	Blinding of participants, personnel and outcome assessors	Incomplete outcome data	Selective outcome reporting	Other sources of bias
Turner, R (UKPDS 33)	Low risk of bias	Low risk of bias	Low risk of bias	Low risk of bias	Low risk of bias	Low risk of bias
Turner, R (UKPDS 34)	Low risk of bias	Low risk of bias	Low risk of bias	Low risk of bias	Low risk of bias	Low risk of bias
Dormandy, HA	Low risk of bias	Low risk of bias	Low risk of bias	Low risk of bias	Low risk of bias	Low risk of bias
Mazzone, T	Low risk of bias	Unclear risk of bias	Low risk of bias	Low risk of bias	High risk of bias	High risk of bias
Nauck, MA	Unclear risk of bias	Unclear risk of bias	Low risk of bias	High risk of bias	Unclear risk of bias	Low risk of bias
Kahn, SE	Low risk of bias	Unclear risk of bias	Low risk of bias	Low risk of bias	Low risk of bias	Low risk of bias
Dargie, HJ	Low risk of bias	Low risk of bias	Low risk of bias	Low risk of bias	Low risk of bias	Low risk of bias
Chan, JC	Unclear risk of bias	Unclear risk of bias	Low risk of bias	High risk of bias	Low risk of bias	High risk of bias
Patel, A (ADVANCE)	Low risk of bias	Low risk of bias	High risk of bias	Low risk of bias	Low risk of bias	Low risk of bias
Holman, RR (4T)	Low risk of bias	Low risk of bias	Low risk of bias	High risk of bias	Low risk of bias	Low risk of bias
Home, PD	Low risk of bias	Low risk of bias	High risk of bias	Low risk of bias	Low risk of bias	High risk of bias
Kooy, A	Low risk of bias	Low risk of bias	Low risk of bias	Low risk of bias	Low risk of bias	Low risk of bias
Bertrand, OF	Unclear risk of bias	Low risk of bias	Low risk of bias	Low risk of bias	Low risk of bias	Low risk of bias
Gaziano, JM	Unclear risk of bias	Unclear risk of bias	Low risk of bias	Low risk of bias	High risk of bias	Low risk of bias
Giles, TD	Unclear risk of bias	Unclear risk of bias	Low risk of bias	Low risk of bias	Low risk of bias	Low risk of bias
Matthews, DR	Unclear risk of bias	Unclear risk of bias	Low risk of bias	Unclear risk of bias	Unclear risk of bias	Low risk of bias
Gallwitz, B (EUREXA)	Low risk of bias	Low risk of bias	Low risk of bias	Low risk of bias	Unclear risk of bias	Low risk of bias
Gallwitz, B (linagliptin)	Low risk of bias	Low risk of bias	Low risk of bias	Low risk of bias	Low risk of bias	Low risk of bias
Zinman, B	Low risk of bias	Low risk of bias	Low risk of bias	Unclear risk of bias	Unclear risk of bias	High risk of bias
Garber, AJ	Low risk of bias	Low risk of bias	Low risk of bias	Low risk of bias	Unclear risk of bias	High risk of bias
Gerstein, HC (ORIGIN)	Low risk of bias	Low risk of bias	High risk of bias	Low risk of bias	Low risk of bias	Low risk of bias

Hong, J	Low risk of bias	Low risk of bias	Low risk of bias	Low risk of bias	Low risk of bias	Low risk of bias
Scirica, BM	Low risk of bias	Low risk of bias	Low risk of bias	Low risk of bias	Low risk of bias	Low risk of bias
White, WB	Low risk of bias	Low risk of bias	Low risk of bias	Unclear risk of bias	Low risk of bias	Low risk of bias
Cefalu, WT	Low risk of bias	Low risk of bias	Low risk of bias	Low risk of bias	Unclear risk of bias	Low risk of bias
Ridderstrale, M	Low risk of bias	Low risk of bias	Low risk of bias	Low risk of bias	Unclear risk of bias	Low risk of bias
Pfeffer, MA	Low risk of bias	Low risk of bias	Low risk of bias	Unclear risk of bias	Low risk of bias	Low risk of bias
Green, JB	Low risk of bias	Low risk of bias	Low risk of bias	Low risk of bias	Low risk of bias	Low risk of bias
Zinman, B (EMPA-REG)	Low risk of bias	Low risk of bias	Low risk of bias	Low risk of bias	Low risk of bias	Low risk of bias
Giorgino, F	Low risk of bias	Low risk of bias	Low risk of bias	Low risk of bias	Low risk of bias	High risk of bias
Blonde, L	Low risk of bias	Low risk of bias	Low risk of bias	High risk of bias	Unclear risk of bias	High risk of bias
Marso, SP (LEADER)	Unclear risk of bias	Unclear risk of bias	Low risk of bias	Low risk of bias	Low risk of bias	Low risk of bias
Marso, SP (SUSTAIN-6)	Unclear risk of bias	Unclear risk of bias	Low risk of bias	Low risk of bias	Low risk of bias	Low risk of bias
Holman, RR (EXSCEL)	Low risk of bias	Low risk of bias	Low risk of bias	Low risk of bias	Low risk of bias	Low risk of bias
Marso, SP (DEVOTE)	Low risk of bias	Low risk of bias	Low risk of bias	Low risk of bias	Low risk of bias	High risk of bias
Neal, B	Low risk of bias	Low risk of bias	Low risk of bias	Low risk of bias	Low risk of bias	Low risk of bias
Vaccaro, O	Unclear risk of bias	Unclear risk of bias	High risk of bias	Low risk of bias	Low risk of bias	Low risk of bias
Rosenstock, J	Low risk of bias	Low risk of bias	Low risk of bias	Low risk of bias	Low risk of bias	Low risk of bias
Hernndez, AF	Low risk of bias	Low risk of bias	Low risk of bias	Low risk of bias	Low risk of bias	Low risk of bias
Pratley, R	Low risk of bias	Low risk of bias	Low risk of bias	High risk of bias	Low risk of bias	Low risk of bias
Wiviott, SD	Low risk of bias	Low risk of bias	Low risk of bias	Low risk of bias	Low risk of bias	Low risk of bias
Gerstein, HC (REWIND)	Low risk of bias	Low risk of bias	Low risk of bias	Low risk of bias	Low risk of bias	Low risk of bias
Perkovic, V	Low risk of bias	Low risk of bias	Low risk of bias	Low risk of bias	Low risk of bias	Low risk of bias
Husain, M	Low risk of bias	Low risk of bias	Low risk of bias	Low risk of bias	Low risk of bias	Low risk of bias
Pieber, TR	Low risk of bias	Low risk of bias	High risk of bias	High risk of bias	Low risk of bias	Low risk of bias
Rosenstock, J (CAROLINA)	Low risk of bias	Low risk of bias	Low risk of bias	Low risk of bias	Low risk of bias	Low risk of bias

Table S4. Detailed analyses results.

Outcome	Factor	RR	95% C.I.	p for heterogeneity	I2	p in binary metaregression	Studies	Patients
All-cause mortality	Overall	0.937	(0.906 - 0.970)	0.307	8.60%		46	216575
	No HbA1c reduction	0.971	(0.923 - 1.021)	0.853	0%	0.138	26	80753
	HbA1c reduction	**0.899**	**(0.844 - 0.958)**	0.081	34.40%		17	103032
	No severe hypoglycemia reduction	0.953	(0.893 - 1.018)	0.101	34.30%	0.143	14	93202
	Severe hypoglycemia reduction	**0.903**	**(0.862 - 0.946)**	0.697	0.00%		21	94244
	No weight reduction	**0.938**	**(0.890 - 0.988)**	0.179	20.20%	0.586	27	118338
	Weight reduction	**0.917**	**(0.868 - 0.968)**	0.579	0.00%		17	72426
	No systolic blood pressure reduction	**0.920**	**(0.852 - 0.992)**	0.089	35.80%	0.642	15	66562
	Systolic blood pressure reduction	**0.903**	**(0.858 - 0.951)**	0.441	0.90%		15	80623
	Insulin-sensitizer	0.934	(0.831 - 1.050)	0.899	0.00%	0.993	11	20226
	Insulin / secretagogue	0.973	(0.917 - 1.032)	0.575	0.00%	0.984	7	36189
	Incretin	**0.937**	**(0.893 - 0.982)**	0.301	8.20%	0.315	21	115372
	SGLT2i	0.875	(0.747 - 1.026)	0.011	66.60%	0.256	6	41718
Cardiovascular mortality	Overall	0.927	(0.881 - 0.976)	0.125	22.50%		35	191796
	No HbA1c reduction	0.975	(0.907 - 1.049)	0.625	0.00%	0.277	16	61212
	HbA1c reduction	**0.880**	**(0.804 - 0.962)**	0.058	39.50%		15	97794
	No severe hypoglycemia reduction	0.956	(0.895 - 1.021)	0.291	16.60%	0.318	10	84096
	Severe hypoglycemia reduction	**0.898**	**(0.825 - 0.978)**	0.155	26.70%		17	88449
	No weight reduction	**0.909**	**(0.838 - 0.987)**	0.075	35.30%	0.970	20	101882
	Weight reduction	0.926	(0.851 - 1.008)	0.359	8.60%		13	64103
	No systolic blood pressure reduction	0.916	(0.821 - 1.023)	0.040	47.30%	0.725	13	64970

	Systolic blood pressure reduction	**0.899**	**(0.826 - 0.978)**	0.232	21.60%		11	73581
	Insulin-sensitizer	0.917	(0.640 - 1.314)	0.371	7.20%	0.874	8	9344
	Insulin / secretagogue	0.972	(0.901 - 1.048)	0.767	0.00%	0.393	5	33336
	Incretin	**0.933**	**(0.883 - 0.986)**	0.609	0.00%	0.907	18	110393
	SGLT2i	0.857	(0.674 - 1.090)	0.001	82.20%	0.336	4	38723
Myocardial infarction	Overall	0.942	(0.907 - 0.979)	0.590	0.00%		34	200150
	No HbA1c reduction	0.952	(0.883 - 1.026)	0.726	0.00%	0.638	16	65054
	HbA1c reduction	**0.928**	**(0.875 - 0.983)**	0.306	12.90%		15	102306
	No severe hypoglycemia reduction	0.973	(0.921 - 1.029)	0.748	0.00%	0.131	12	91693
	Severe hypoglycemia reduction	**0.914**	**(0.861 - 0.970)**	0.421	2.80%		16	88916
	No weight reduction	0.948	(0.893 - 1.007)	0.252	16.20%	0.550	20	107389
	Weight reduction	**0.923**	**(0.859 - 0.991)**	0.754	0.00%		12	66950
	No systolic blood pressure reduction	0.960	(0.879 - 1.048)	0.180	26.90%	0.503	12	64571
	Systolic blood pressure reduction	**0.927**	**(0.869 - 0.989)**	0.534	0.00%		12	73227
	Insulin-sensitizer	0.889	(0.765 - 1.031)	0.871	0.00%	0.429	9	19702
	Insulin / secretagogue	0.947	(0.876 - 1.023)	0.470	0.00%	0.451	5	34167
	Incretin	0.954	(0.898 - 1.014)	0.264	16.20%	0.294	16	108889
	SGLT2i	0.925	(0.840 - 1.019)	0.571	0.00%	0.691	3	34322
Stroke	Overall	0.934	(0.885 - 0.986)	0.354	6.90%		34	200942
	No HbA1c reduction	0.934	(0.819 - 1.065)	0.157	26.40%	0.551	16	65847
	HbA1c reduction	**0.912**	**(0.847 - 0.982)**	0.463	0.00%		15	102305
	No severe hypoglycemia reduction	0.950	(0.887 - 1.016)	0.666	0.00%	0.451	11	91708
	Severe hypoglycemia reduction	0.913	(0.827 - 1.007)	0.279	16.00%		16	89693
	No weight reduction	0.957	(0.881 - 1.039)	0.332	10.10%	0.294	20	108182
	Weight reduction	**0.887**	**(0.800 - 0.984)**	0.320	12.40%		12	66949
	No systolic blood pressure reduction	0.964	(0.881 - 1.055)	0.709	0.00%	0.280	13	65455

	Systolic blood pressure reduction	**0.889**	**(0.815 - 0.970)**	0.430	1.40%		12	73226
	Insulin-sensitizer	**0.787**	**(0.658 - 0.940)**	0.901	0.00%	0.055	9	19702
	Insulin / secretagogue	0.994	(0.893 - 1.106)	0.302	17.70%	0.176	5	34167
	Incretin	**0.898**	**(0.825 - 0.977)**	0.368	7.70%	0.135	16	109681
	SGLT2i	1.051	(0.926 - 1.193)	0.641	0.00%	0.064	3	34322
Heart failure	Overall	0.976	(0.887 - 1.075)	0.001	68.10%		31	178741
	No HbA1c reduction	1.102	(0.907 - 1.339)	0.001	60.90%	0.167	16	58681
	HbA1c reduction	0.916	(0.780 - 1.075)	0.001	79.90%		12	87270
	No severe hypoglycemia reduction	1.099	(0.948 - 1.274)	0.001	75.10%	0.060	11	86328
	Severe hypoglycemia reduction	**0.891**	**(0.797 - 0.996)**	0.069	42.00%		13	68364
	No weight reduction	1.032	(0.873 - 1.221)	0.001	77.90%	0.401	17	85980
	Weight reduction	**0.897**	**(0.810 - 0.994)**	0.247	20.00%		12	66950
	No systolic blood pressure reduction	1.002	(0.837 - 1.201)	0.003	66.20%	0.156	10	45434
	Systolic blood pressure reduction	**0.843**	**(0.769 - 0.925)**	0.259	18.90%		13	77628
	Insulin-sensitizer	**1.433**	**(1.211 - 1.695)**	0.387	5.70%	0.001	7	15488
	Insulin / secretagogue	0.937	(0.857 - 1.025)	0.434	0.00%	0.865	4	26530
	Incretin	0.991	(0.921 - 1.065)	0.376	7.10%	0.674	15	94930
	SGLT2i	**0.709**	**(0.633 - 0.793)**	0.630	0.00%	0.001	4	38723

Table S5. Summary of Findings Table

Antihyperglycemic agents compared to control for type 2 diabetes

Patient or population: type 2 diabetes
Setting: Effects on metabolic factors (glycemic control, hypoglycemia, weight and systolic blood pressure).
Intervention: antihyperglycemic agents
Comparison: control

Outcomes	Anticipated absolute effects* (95% CI)		Relative effect (95% CI)	№ of participants (studies)	Certainty of the evidence (GRADE)	Comments
	**Risk with control**	**Risk with antihyperglycemic agents**				
All-cause mortality according to metabolic factors follow up: mean 3.5 years	74 per 1.000	**69 per 1.000** (67 to 72)	**RR 0.937** (0.906 to 0.970)	216575 (46 RCTs)	⊕⊕⊕◯ MODERATE [a]	Antihyperglycemic agent probably results in a slight reduction in all-cause mortality risk according to glycemic control, severe hypoglycemia risk, and systolic blood pressure.
Cardiovascular mortality according to metabolic factors follow up: median 3.1 years	49 per 1.000	**45 per 1.000** (43 to 48)	**RR 0.927** (0.881 to 0.976)	191796 (35 RCTs)	⊕⊕⊕◯ MODERATE [a]	Antihyperglycemic agent probably results in a slight reduction in cardiovascular mortality risk according to glycemic control, severe hypoglycemia risk, and systolic blood pressure.
Myocardial infarction according to metabolic factors follow up: mean 4.1 years	52 per 1.000	**49 per 1.000** (47 to 51)	**RR 0.942** (0.907 to 0.979)	200150 (34 RCTs)	⊕⊕⊕◯ MODERATE [a]	Antihyperglycemic agent probably results in a slight reduction in myocardial infarction risk according to glycemic control, severe hypoglycemia risk, body weight, and systolic blood pressure.
Stroke according to metabolic factors follow up: mean 3.8 years	32 per 1.000	**30 per 1.000** (28 to 31)	**RR 0.934** (0.885 to 0.986)	200942 (34 RCTs)	⊕⊕⊕◯ MODERATE [a]	Antihyperglycemic agent probably results in a slight reduction in stroke risk according to glycemic control, and body weight.
Heart failure according to metabolic factors follow up: mean 3.6 years	42 per 1.000	**41 per 1.000** (38 to 46)	**RR 0.976** (0.887 to 1.075)	178741 (31 RCTs)	⊕⊕⊕◯ MODERATE [a,b]	Antihyperglycemic agent probably results in a slight reduction in heart failure according to severe hypoglycemia risk, body weight, and systolic blood pressure.

***The risk in the intervention group** (and its 95% confidence interval) is based on the assumed risk in the comparison group and the **relative effect** of the intervention (and its 95% CI).

CI: Confidence interval; **RR:** Risk ratio

Explanations

a. Subgroup analysis are considered indirect.
b. Elevated statistical heterogeneity

(A) All-cause mortality by mechanism of action.

All–cause Mortality

Variable	p Meta–regression	RR	95% C.I.	Number of patients
**Overall**		**0.937**	**0.906 – 0.970**	**216575**
Sensitizers	0.993	0.934	0.831 – 1.050	20226
Insulin / secretagogues	0.984	0.973	0.917 – 1.032	36189
Incretins	0.315	0.937	0.893 – 0.982	115372
SGLT2i	0.256	0.875	0.747 – 1.026	41718

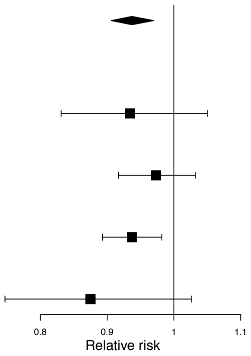

(B) Cardiovascular mortality by mechanism of action.

Cardiovascular Mortality

Variable	p Meta–regression	RR	95% C.I.	Number of patients
**Overall**		**0.927**	**0.881 – 0.976**	**191796**
Sensitizers	0.874	0.917	0.640 – 1.314	9344
Insulin / secretagogues	0.393	0.972	0.901 – 1.048	33336
Incretins	0.907	0.933	0.883 – 0.986	110393
SGLT2i	0.336	0.857	0.674 – 1.090	38723

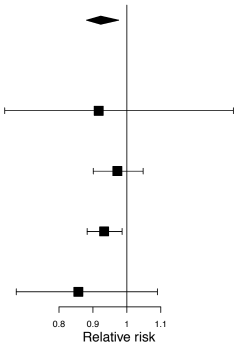

(C) Myocardial infarction by metabolic factors.

Myocardial Infarction

Variable	p Meta–regression	RR	95% C.I.	Number of patients
**Overall**		**0.942**	**0.907 – 0.979**	**200150**
HbA1c				
No reduction	0.638	0.952	0.883 – 1.026	65054
Reduction		0.928	0.875 – 0.983	102306
Severe hypoglycemia				
No reduction	0.131	0.973	0.921 – 1.029	91693
Reduction		0.914	0.861 – 0.970	88916
Weight				
No reduction	0.550	0.948	0.893 – 1.007	107389
Reduction		0.923	0.859 – 0.911	66950
Systolic blood pressure				
No reduction	0.503	0.960	0.879 – 1.048	64571
Reduction		0.927	0.869 – 0.989	73227

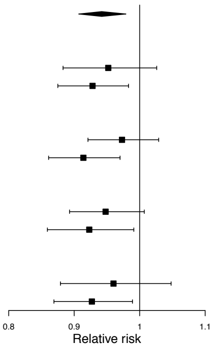

(D) Myocardial infarction by mechanism of action.

Myocardial Infarction

Variable	p Meta–regression	RR	95% C.I.	Number of patients
**Overall**		**0.942**	**0.907 – 0.979**	**200150**
Sensitizers	0.429	0.889	0.765 – 1.031	19702
Insulin / secretagogues	0.885	0.947	0.876 – 1.023	34167
Incretins	0.487	0.954	0.898 – 1.014	108889
SGLT2i	0.691	0.925	0.840 – 1.019	34322

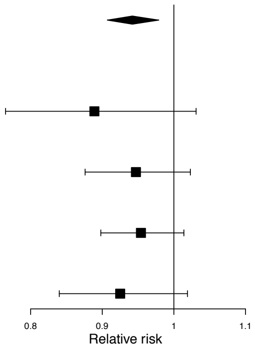

(E) Stroke by metabolic factors.

Stroke

Variable	p Meta–regression	RR	95% C.I.	Number of patients
**Overall**		**0.934**	**0.885 – 0.986**	**200942**
HbA1c				
No reduction	0.551	0.934	0.819 – 1.065	65847
Reduction		0.912	0.847 – 0.982	102305
Severe hypoglycemia				
No reduction	0.451	0.950	0.887 – 1.016	91708
Reduction		0.913	0.827 – 1.007	89693
Weight				
No reduction	0.294	0.957	0.881 – 1.039	108182
Reduction		0.887	0.800 – 0.984	66949
Systolic blood pressure				
No reduction	0.280	0.964	0.881 – 1.055	65455
Reduction		0.889	0.815 – 0.970	73226

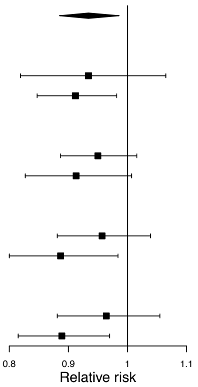

(F) Stroke by mechanism of action.

Stroke

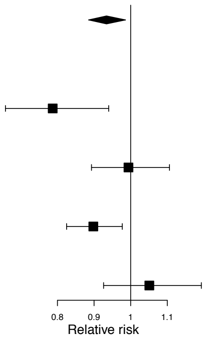

Variable	p Meta−regression	RR	95% C.I.	Number of patients
**Overall**		**0.934**	**0.885 – 0.986**	**200942**
Sensitizers	0.055	0.787	0.658 – 0.940	19702
Insulin / secretagogues	0.176	0.994	0.893 – 1.106	34167
Incretins	0.135	0.898	0.825 – 0.977	109681
SGLT2i	0.064	1.051	0.926 – 1.193	34322

(G) Heart failure by metabolic factors.

Heart Failure

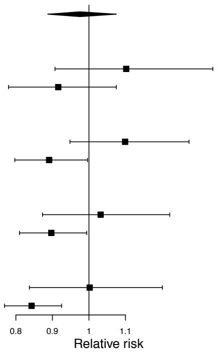

Variable	p Meta−regression	RR	95% C.I.	Number of patients
**Overall**		**0.976**	**0.887 – 1.075**	**178741**
HbA1c				
No reduction	0.167	1.102	0.907 – 1.339	58681
Reduction		0.916	0.780 – 1.075	87270
Severe hypoglycemia				
No reduction	0.060	1.099	0.948 – 1.274	86328
Reduction		0.891	0.797 – 0.996	68364
Weight				
No reduction	0.401	1.032	0.873 – 1.221	85980
Reduction		0.897	0.810 – 0.994	66950
Systolic blood pressure				
No reduction	0.156	1.002	0.837 – 1.201	45434
Reduction		0.843	0.769 – 0.925	77628

(H) Heart failure by mechanism of action.

Heart Failure

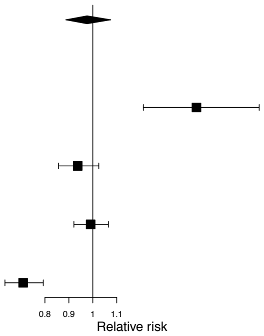

Variable	p Meta−regression	RR	95% C.I.	Number of patients
**Overall**		**0.976**	**0.887 – 1.075**	**178741**
Sensitizers	0.9	1.433	1.211 – 1.695	15488
Insulin / secretagogues	0.9	0.937	0.857 – 1.025	26530
Incretins	0.9	0.991	0.921 – 1.065	94930
SGLT2i	0.9	0.709	0.633 – 0.793	38723

Figure S1. Forest plot for antihyperglycemic agents and all-cause and cardiovascular mortality relative risks according to metabolic factors or mechanism of action. Legend: (A) all-cause mortality by mechanism of action; (B) cardiovascular mortality by mechanism of action; (C) myocardial infarction by metabolic factors; (D) myocardial infarction by mechanism of action; (E) stroke by metabolic factors; (F) stroke by mechanism of action; (G) heart failure by metabolic factors; (H) heart failure by mechanism of action.

(A) All-cause mortality

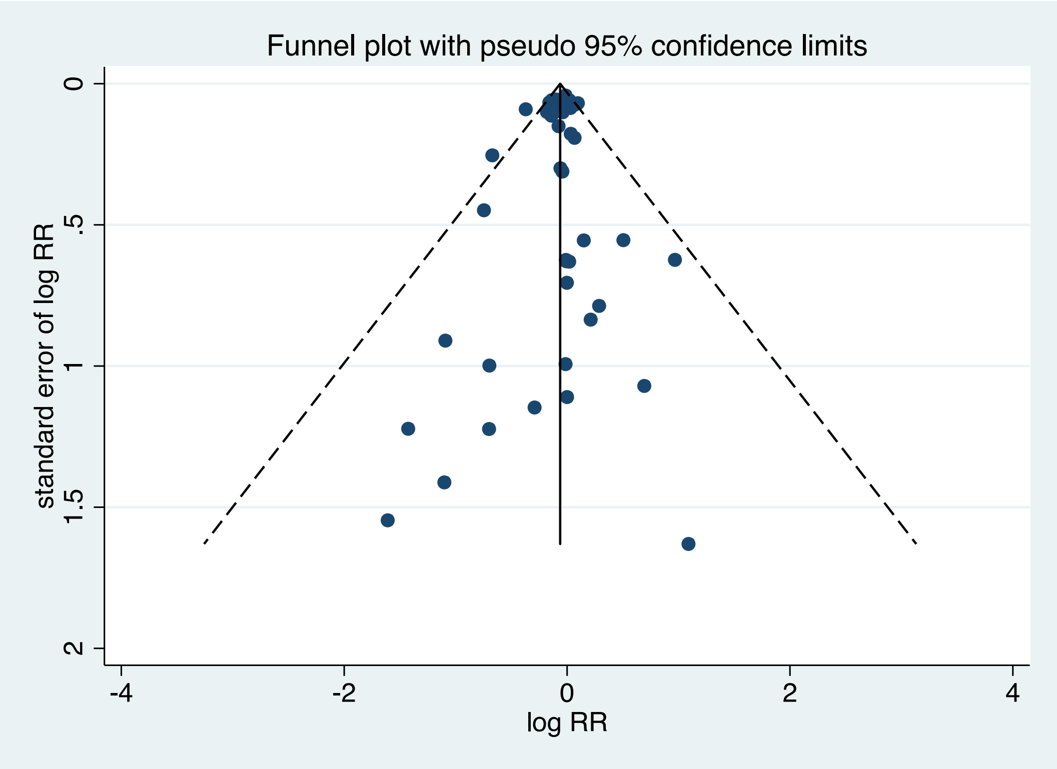

(B) Cardiovascular mortality

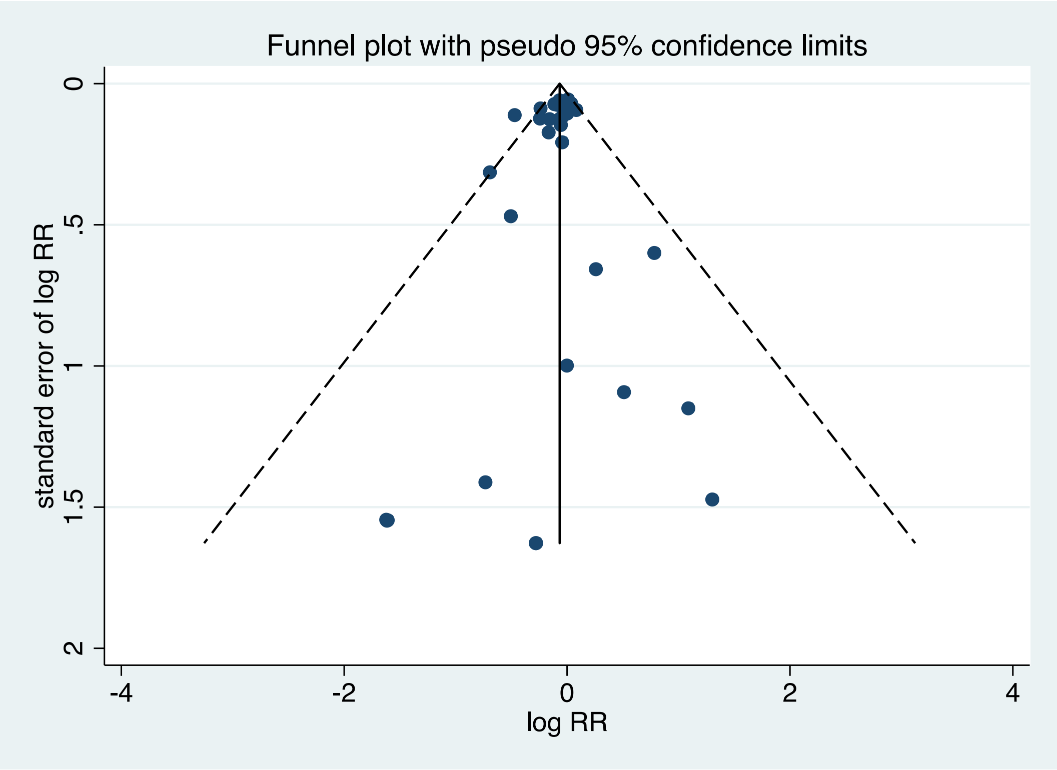

(C) Myocardial infarction

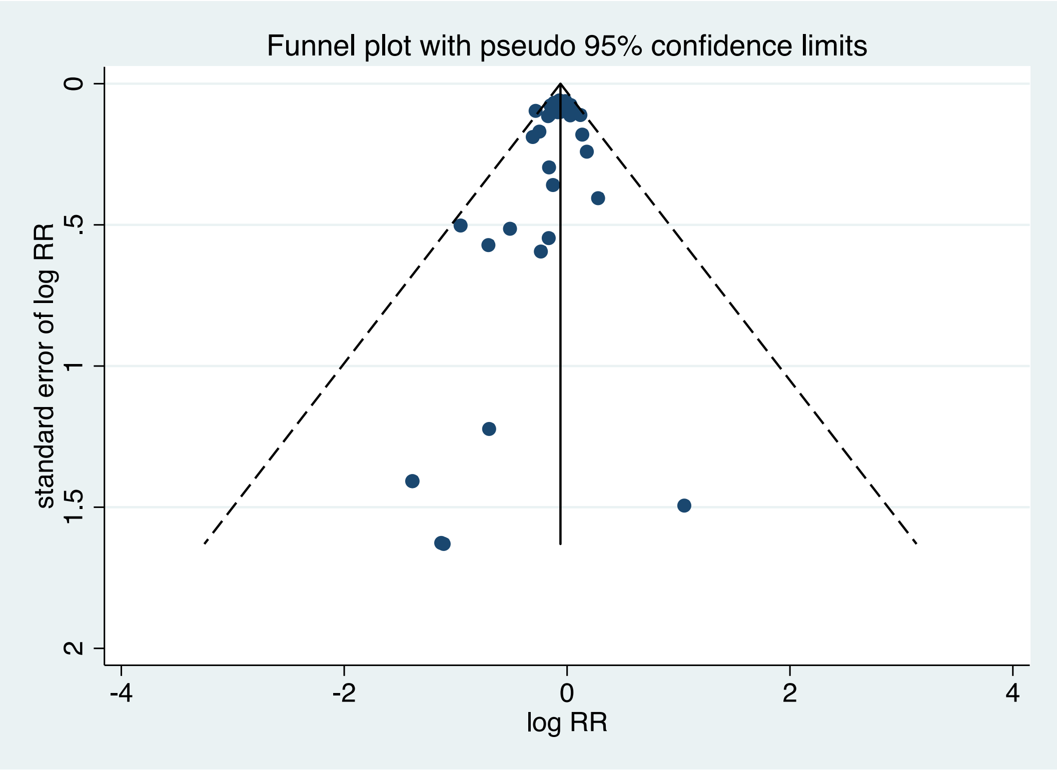

(D) Stroke

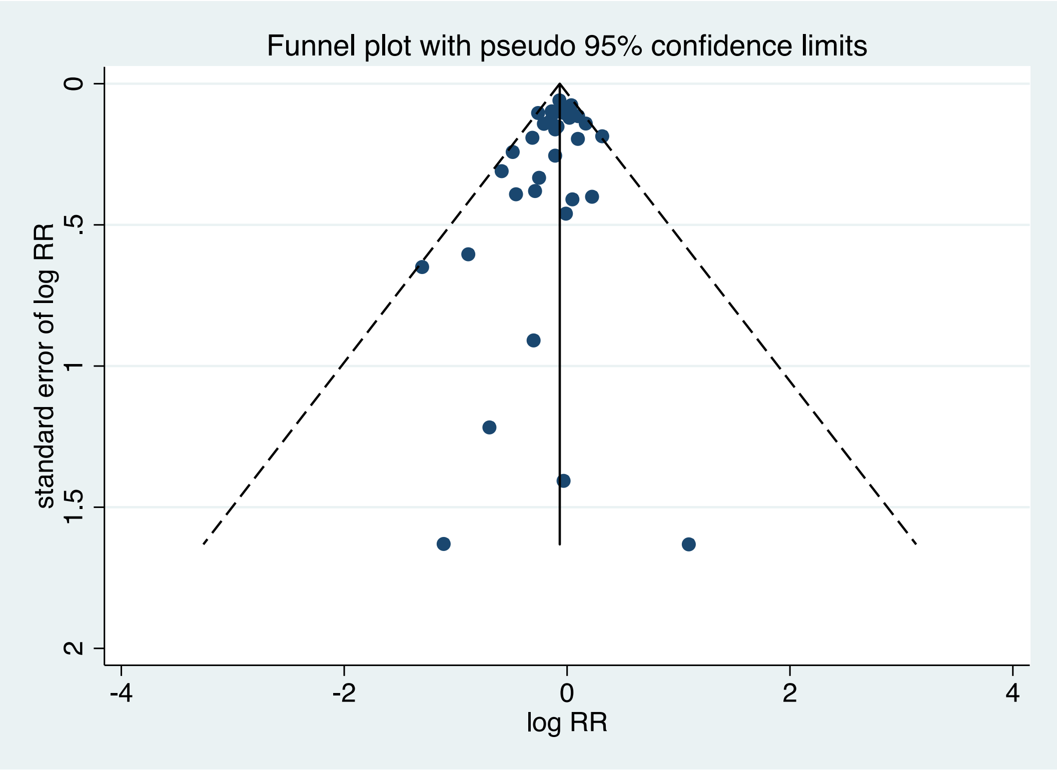

(E) Heart failure

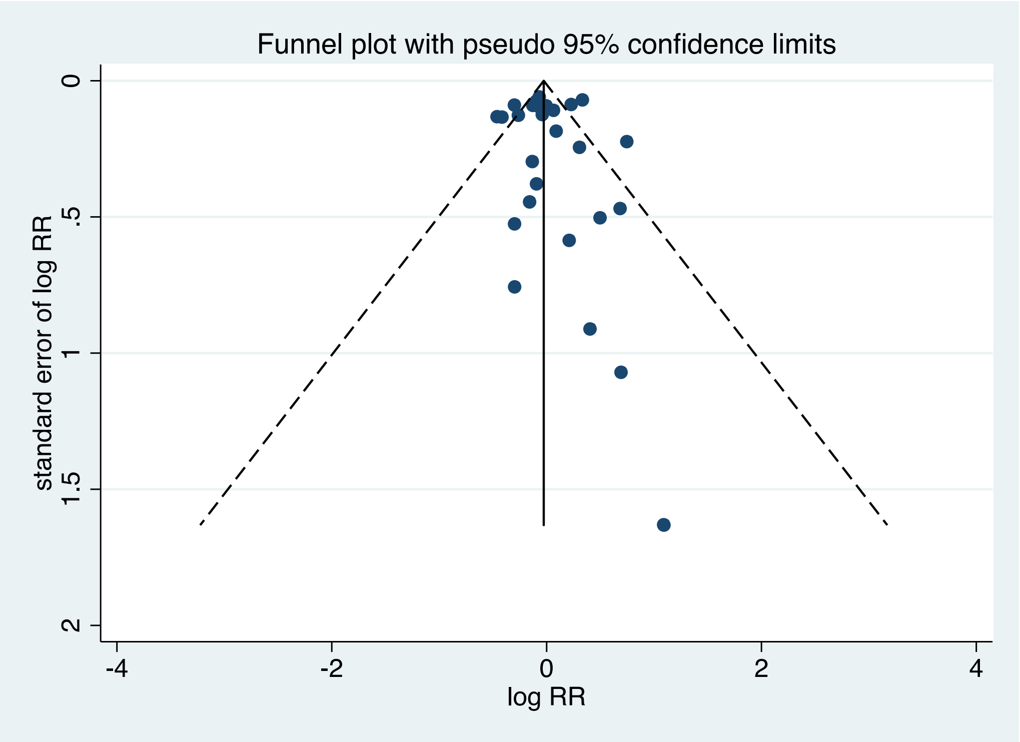

Figure S2. Funnel plots for (A) all-cause mortality, (B) cardiovascular mortality, (C) Myocardial infarction, (D) stroke, (E) heart failure.
